# Microsomal triglyceride transfer protein gene -493G/T polymorphism and its association with serum lipid levels in Bama Zhuang long-living families in China

**DOI:** 10.1186/1476-511X-11-177

**Published:** 2012-12-28

**Authors:** Shang-Ling Pan, Xiao-Qiu Luo, Ze-Ping Lu, Shao-Hua Lu, Huan Luo, Cheng-Wu Liu, Cai-You Hu, Ming Yang, Li-Li Du, Zhen Song, Guo-Fang Pang, Hua-Yu Wu, Jin-Bo Huang, Jun-Hua Peng, Rui-Xing Yin

**Affiliations:** 1 Department of Cardiology, Institute of Cardiovascular Diseases, the First Affiliated Hospital, Guangxi Medical University, 22 Shuangyong Road, Nanning, Guangxi, 530021, People’s Republic of China; 2 Department of Pathophysiology, School of Premedical Sciences, Guangxi Medical University, Nanning, Guangxi, 530021, People’s Republic of China; 3 Department of Neurology, Jiangbin Hospital of Guangxi Zhuang Autonomous Region, 85 Hedi Road, Nanning, Guangxi, 530021, People’s Republic of China; 4 Department of Cell Biology & Genetics, School of Premedical Sciences, Guangxi Medical University, Nanning, Guangxi, 530021, People’s Republic of China

## Abstract

**Background:**

The -493G/T polymorphism in the microsomal triglyceride transfer protein (MTP) gene is associated with lower serum low-density lipoprotein cholesterol (LDL-C) and triglyceride (TG) levels and longevity in several populations, but the results are inconsistent in different racial/ethnic groups. The current study was to investigate the plausible association of MTP -493G/T polymorphism with serum lipid levels and longevity in Zhuang long-lived families residing in Bama area, a famous home of longevity in Guangxi, China.

**Methods:**

The MTP -493G/T was genotyped by PCR-restriction fragment length polymorphism in 391 Bama Zhuang long-lived families (BLF, n = 1467, age 56.60 ± 29.43 years) and four control groups recruited from Bama and out-of-Bama area with or without a familial history of exceptional longevity: Bama non-long-lived families (BNLF, n = 586, age 44.81 ± 26.83 years), Bama non-Zhuang long-lived families (BNZLF, n = 444, age 52.09 ± 31.91 years), Pingguo long-lived families (PLF, n = 658, age 50.83 ± 30.30 years), and Pingguo non-long-lived families (PNLF, n = 539, age 38.74 ± 24.69 years). Correlation analyses between genotypes and serum lipid levels and longevity were then performed.

**Results:**

No particularly favorable lipoprotein and clinical phenotypes were seen in BLF as compared to general families in the same area. Instead, the levels of total cholesterol (TC), TG, LDL-C, and the prevalence of dyslipidemia were significantly higher in the three Bama families as compared to the two non-Bama families (*P* < 0.01 for all). There were no differences in the allelic and genotypic frequencies among the tested cohorts (*P* > 0.05 for all), but the TT genotype tended to enrich in the three long-lived cohorts from both areas. In addition, the individuals harboring TT genotype exhibited lower LDL-C and TC levels in the overall populations and Bama populations with a region- and sex-specific pattern. Multiple linear regression analyses unraveled that LDL-C levels were correlated with genotypes in Bama combined population, BNLF, and the total population (*P* < 0.05 for each) but not in Pingguo populations; TC and HDL-C levels were correlated with genotypes in Bama combined population and BLF, respectively (*P* < 0.05 for each).

**Conclusions:**

MTP -493G/T polymorphism may play an important role in fashioning the serum lipid profiles of Bama populations, despite no direct association between MTP -493G/T and longevity was detected.

## Introduction

A number of epidemiological studies have demonstrated that the elevation in plasma cholesterol is a key and independent risk factor in the etiology of coronary heart disease (CHD) which may further influence human mortality, aging, and lifespan [1-3]. The body cholesterol homeostasis is believed to be related to the individual’s genetic background and various environmental factors [4]. Microsomal triglyceride transfer protein (MTP, or MTTP) is a heterodimeric lipid transfer protein mainly present in hepatocytes, enterocytes and myocytes which transfers lipid to a nascent apolipoprotein B (apoB) molecule when it enters the lumen of the endoplasmic reticulum (ER), making it an indispensable genetic component in the maintenance of body cholesterol balance via pathways of absorption, synthesis, transfer, or secretion [2]. Recently, MTP gene has emerged as an appealing candidate gene involving in human longevity as its functions and essential position in lipoprotein assembly resemble apoE, the most extensively studied and consistently replicated gene affecting human longevity [5]. In this context, there are reasons to assume that functional variants in the MTP gene may modulate MTP concentration or activity, affect plasma lipid levels and therefore contribute to the development of atherosclerosis, CHD and aging, some of which have proved to be true [6,7]. To date, several variants on MTP gene have been detected (e.g. -285G/C, -383T/C, -891C/G, -164T/C, -400A/T and -493G/T), of which the −493G/T single nucleotide polymorphism (SNP) in the promoter region (rs1800591, chromosome 4, contig NT 016354.18 position 25043208) has been mostly studied due to its plausible role in the modulation of lipid/lipoprotein profiles [8]. Most investigators reported an association between the MTP -493T allele and low levels of serum total cholesterol (TC), low-density lipoprotein cholesterol (LDL-C), and apoB [9-13], whereas others have either revealed an association of -493T allele with increased concentrations of TC, LDL-C, triglyceride (TG) and apoB [14-17] or detected no relationship between this polymorphism and any lipid phenotype [18]. Similar contradictory data have also been obtained in the association studies between the -493G/T MTP polymorphism and human longevity, in which the “no association hypothesis” appears to be increasingly overwhelming [3,19-21]. Collectively, there are no last words on whether or to what extent MTP −493 polymorphisms associate with lipoprotein profiles and human longevity. The underlying reasons for the discrepancy in these findings might relate to differences in the populations studied or might be confounded by mixture or population stratification in control participants [18,22]. Thus, replication studies in large and well-defined samples from different populations have been emphasizing [19]. Indeed, it is worth noting that the available data in this research subfield are primarily based on Caucasian populations, observations from eastern ethnic groups, especially from minorities in China, are limited.

Zhuang is the largest minority in China and mainly lives in the northwest of Guangxi province, with a population of around 15 millions. Due to a long-term isolation by huge mountains or rivers over the past millennia, Zhuangs have differentiated into several distinct branches with significant heterogeneity in culture, custom, lifestyle, dietary habit, morbility and mortality [23-25]. For instance, the Yongnan Zhuangs residing in Fusui, a county along the southern bank of Yongjiang River, are prone to liver cancer which contributes to a higher mortality [26], whereas Bama Zhuangs inhabiting the Honghuihe River Basin have often been spared from major age-related diseases and have prolonged lifespan [27-29]. As such, together with its lower genetic background, Bama Zhuang has become a unique and useful population for human longevity study over the past decades [30,31]. Major efforts have been made for identifying the environmental and genetic factors involved in longevity in the region, but no precise mechanisms has been established. Whether MTP the −493 polymorphism is an important predictor of longevity in Bama population remains to be determined.

The current study is a part of the ongoing Bama Longevity Genetic Study (BLGS) which was undertaken from 2008 to 2011 as described elsewhere [30]. We hypothesized that Bama Zhuang long-lived families might have unique lipid profiles in relation to their favorable MTP −493 genotypes, which ultimately accounts in part for the longevity in the area. To that aim, we characterized the prevalence of SNP rs1800591 and performed association analysis in Bama Zhuang long-lived families and well-organized control families with or without a history of exceptional longevity.

## Materials and methods

### Study design and participants

The individuals investigated in the current study participate in the ongoing BLGS [30]. Long-lived Families participating in the BLGS (Bama long-lived families, BLF) have at least two siblings fulfilling four inclusion criteria: (i) aged 90 or above, (ii) participants have one or more living brother or sister who satisfy the first criterion, (iii) the nonagenarian sibship has identical parents, (iv) parents of the nonagenarians belong to Hongshuihe Zhuang branch. Here, the BLF included 1467 members (817 males and 650 females, age 56.60 ± 29.43 and range 3–107 years) belonging to 391 sibships residing in Bama area (Bama, Fengshan, Donglan, and Du’an County) along the midstream of Hongshuihe River Basin, Guangxi Zhuang Autonomous Region, the People’s Republic of China.

We used four different referent groups in the current study, two of which are from Bama area (environmental-matched), and the two others are from Pingguo, a county belonging to another water system, Youjiang River, which is approximately 200 kilometers away from Bama area (environmental-unmatched). The first control group, Bama non-long-lived families (BNLF), consisted of 586 members (350 males and 236 females, age 44.81 ± 26.83 and range 4–89 years) belonging to 183 sibships without a history of exceptional longevity recruited from the same area as BLF (assuming that they represent the general population). The second control group, Bama non-Zhuang long-lived families (BNZLF), included 444 members (246 males and 198 females, age 52.09 ± 31.91 and range 6–94 years) belonging to 96 sibships (mainly Han and Yao ethnic group) living in Bama area and meeting the first three criteria of long-lived families as described above. The third control group, Pingguo long-lived families (PLF), consisted of 658 members (390 males and 268 females, age 50.83 ± 30.30 and range 2–103 years) belonging to 144 sibships from long-lived Zhuang families inhabiting in Pingguo County. The forth control group, Pingguo non-long-lived families (PNLF), consisted of 539 members (334 males and 205 females, age 38.74 ± 24.69 and range 2–89 years) belonging to 108 sibships enrolled from general families without a history of exceptional longevity living in Pingguo County. These control families were set in an attempt to attenuate the potential effects of environmental factors such as dietary habit and lifestyle or ethnic background on our observational variables.

The current study was approved by the ethics committee of Guangxi Medical University. All participants or their legally authorized representatives took part in the written informed consent process after receiving a full explanation of the study. The ages of participants were defined by dates of birth as stated on identity cards or birth certificates. Additional data collected included health and socio-demographic histories. All subjects were essentially healthy and had no evidence of any major or chronic illness. Exclusion criteria were diabetes, a history of CHD or arterial thromboembolic disease, and continuous treatment with antihypertensive or lipid-lowering agents.

### Epidemiological survey

Socio-demographic information was obtained using a standardized questionnaire. Anthropometric variables including height, weight and waist were measured in all groups. Body mass index (BMI) was calculated as weight (kg)/height^2^ (m). Sitting blood pressure was measured 3 times, using a standard mercury sphygmomanometer with the subject resting for at least 5 minutes, and the average of the 3 measurements was used for the level of blood pressure. Systolic blood pressure was determined by the first Korotkoff sound; and diastolic, by the fifth Korotkoff sound. Hypertension was defined as systolic blood pressure > 140 mmHg and/or diastolic blood pressure > 90 mmHg [32]. Normal weight, overweight, and obesity were defined as a BMI < 24, 24 to 28, and > 28 kg/m^2^, respectively [33].

### Biochemical analyses

A venous blood sample of 8 mL was drawn from each subject after an overnight fast, 4 mL of which was collected in a glass tube for serum lipid determination, while the remaining 4 mL was transferred to a tube with anticoagulant solution (4.80 g/L citric acid, 14.70 g/L glucose, and 13.20 g/L trisodium citrate) for DNA extraction. The levels TC, TG, LDL-C and high-density lipoprotein cholesterol (HDL-C) in samples were determined by enzymatic methods with commercially available kits, Tcho-1, TGLH (Randox Laboratories Ltd, Crumlin, Antrim, United Kingdom), Cholestest N HDL, and Cholestest LDL (Daiichi Pure Chemicals Co, Ltd., Tokyo, Japan), respectively. All determinations were performed by standard automated methods on a biochemical analyzer (Type 7170A; Hitachi Ltd, Tokyo, Japan) at the Clinical Science Experiment Center of the First Affiliated Hospital, Guangxi Medical University. The normal ranges of serum TC, TG, HDL-C, and LDL-C levels in the center were 3.10-5.17, 0.56-1.70, 0.91-1.81, and 1.70-3.20 mmol/L, respectively. Hyperlipidemia was defined as TC > 5.17 mmol/L and/or TG > 1.70 mmol/L [25].

### Genotyping

**Figure 1:**
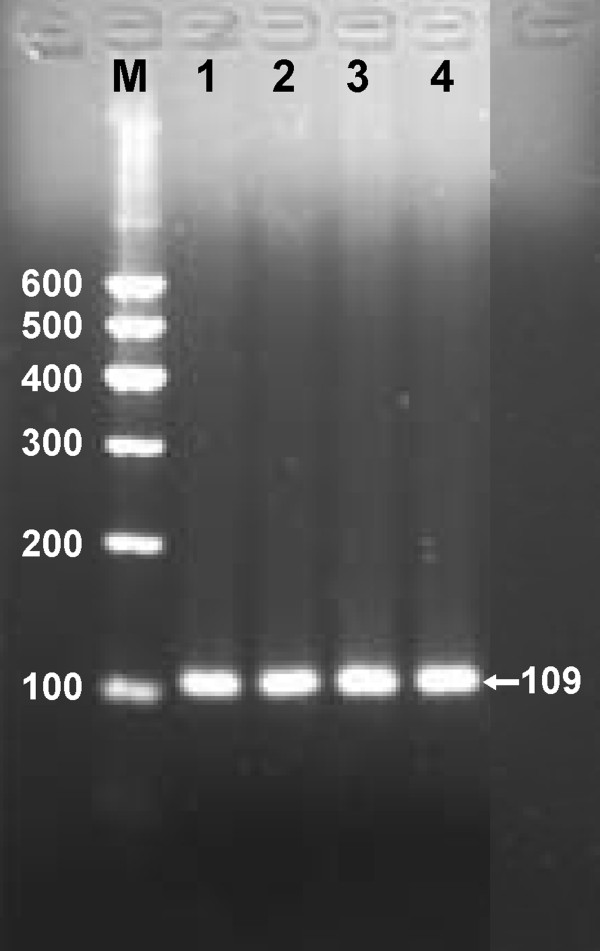
**PCR products of target fragment.** Lane M, 100 bp marker ladder; lanes 1–4, samples. The 109 bp bands are the target fragments.

**Figure 2:**
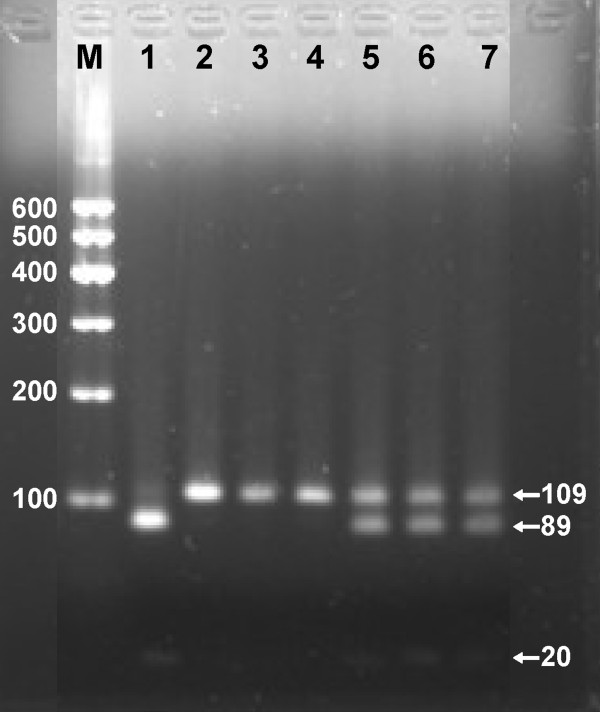
**Genotype results of MTP -493G/T polymorphism.** Lane M, 100 bp marker ladder; lanes 1, GG genotype (89- and 20-bp); lanes 2–4, TT genotype (109 bp); and lanes 5–7, GT genotype (109-, 89- and 20-bp).

**Figure 3:**
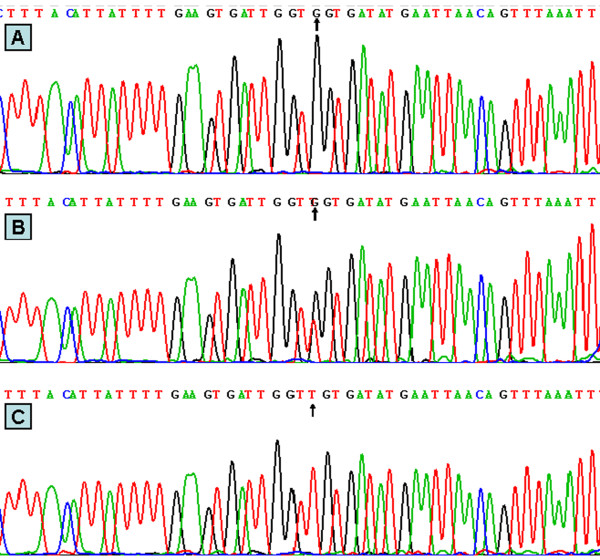
**Partial nucleotide sequences of SNP rs1800591.** (**A**) GG genotype; (**B**) GT genotype; (**C**) TT genotype.

Genomic DNA was prepared from white blood cells by a standard method [34]. The MTP -493G/T polymorphism was genotyped by a polymerase chain reaction (PCR)-restriction fragment length polymorphism assay as described by Karpe et al. [9]. Briefly, a 109 bp fragment in the promoter region of the MTP gene was amplified by PCR, with use of the following primers: F: 5^′^-AGTTTCACACATAAGGACAATCATCTA-3^′^ and R: 5^′^-GGATTTAAATTTAAACTGTTAATTCATATCAC-3^′^ (Sangon Biotech Co., Ltd., Shanghai, People’s Republic of China). PCR was performed in a volume of 20 μL containing 200 ng of genomic DNA, 10 μL of *Taq* MasterMix (Beijing CoWin Bioscience Co., Ltd. People’s Republic of China), 6.25 μM (2.0 μL) of each primer, 5 μL ddH_2_O and 1 U of DNA polymerase (Takara Biotechnology Co, Ltd, Dalian, People’s Republic of China). The cycle profile was as follow: predenaturation at 95°C for 5 min, followed by 30 cycles of denaturation at 95°C for 30 s, annealing at 58°C for 30 s, and extension at 72°C for 30 s, with a final extension at 72°C for 7 min. PCR products (10 μL) were incubated with 2.5 U *Hph*I (New England Biolabs, Beverly, MA, USA) at 37°C for 4 hours, run on 3.5% agarose for 50 min at 120 V, and visualized by staining for 30 min in 5 μg/mL ethidium bromide. A “G” at position −493 yielded bands of 89 and 20 bp, whereas a “T” at position −493 yielded a band of 109 bp, thus individual with band(s) at 89 and 20 bp, at 109 bp only, and at 109, 89 and 20 bp was defined as GG homozygotic genotype, TT homozygotic genotype, and GT heterozygotic genotype, respectively (Figures 1 and 2). Genotypes were randomly confirmed by DNA sequencing (Figure 3). Laboratory technicians who performed genotyping were masked to clinical and biochemical data.

### Statistical analysis

Continuous variables are presented as mean ± SD. Comparisons of mean values of general characteristics between study groups were performed with the student unpaired *t* test. Allelic and genotypic frequencies were calculated directly. Hardy-Weinberg equilibrium was computed for the expected genotype distribution using the standard goodness-of-fit test. Difference in genotype and allele distribution between the groups was estimated by using the chi-square test. The association of MTP genotypes with serum lipid variables was evaluated using analysis of covariance (ANCOVA). In order to assess the association of serum lipid levels with genotypes (TT = 1, GG = 2 and GT = 3) and several environment factors, multivariable linear regression analyses with stepwise modeling were also performed. All tests were two-sided and *P*-values of 0.05 were considered significant. Data were analyzed using the statistical software package SPSS 16.0 (SPSS Inc, Chicago, IL).

## Results

### General characteristics and serum lipid levels

The basic demographic, clinical, and biochemical characteristics of participants from different groups are summarized in Tables 1 and 2. Anthropometrically, the waist circumference in BLF is significantly higher than that in the local general population; BMI was similar between BLF and BNLF, both of which were markedly higher than in PLF but lower than in BNZLF. Higher systolic and diastolic blood pressures were seen in BLF and the two local control groups as compared to non-local control groups. Roughly the same as blood pressure, typical measures of lipoproteins except HDL-C and prevalence of dyslipidemia were also significantly higher in BLF than in the two Pingguo groups, although these measures were quite similar among Bama groups. These trends remained in the nonagenarians stratified from different long-lived families (Tables 1 and 2). Taken together, no particularly favorable lipoprotein and clinical phenotypes was seen as predicted in BLF when compared to general families in the same area. Instead, several lipid indices were found noticeably higher in the three Bama families when compared to the two non-Bama families.

**Table 1:** Clinical characteristics and serum lipid properties according to families

**Parameter**	**BLF**	**BNLF**	**BNZLF**	**PLF**	**PNLF**
Number (male/female)	1467 (817/650)	586 (350/236)	444 (246/198)	658 (390/268)	539 (334/205)
Age (years)	56.60 ± 29.43	44.81 ± 26.83^b^	52.09 ± 31.91^b^	50.83 ± 30.30^b^	38.74 ± 24.69^bd^
Chest (cm)	80.11 ± 21.39	77.58 ± 10.79	80.43 ± 11.38	78.58 ± 12.34	77.14 ± 13.59
Waist (cm)	73.14 ± 15.05	68.33 ± 11.12^b^	73.35 ± 12.11	71.25 ± 12.03	70.48 ± 13.25^d^
Hip (cm)	84.67 ± 9.79	82.94 ± 10.27	84.92 ± 11.58	83.18 ± 13.10	82.71 ± 13.96
BMI (kg/m^2^)	20.69 ± 3.68	20.02 ± 3.65	21.13 ± 4.0^b^	20.01 ± 3.85^b^	20.01 ± 4.31^c^
SBP (mmHg)	143.44 ± 28.08	133.90 ± 25.23	139.52 ± 29.00	137.27 ± 27.37^b^	128.91 ± 22.22^bd^
DBP (mmHg)	86.32 ± 12.52	83.59 ± 12.11	84.76 ± 12.29	81.54 ± 11.42^b^	81.64 ± 11.47^bc^
TC (mmol/L)	5.07 ± 1.03	4.92 ± 0.99	4.90 ± 1.09^a^	4.39 ± 0.96^b^	4.36 ± 1.01^bd^
TG (mmol/L)	1.29 ± 0.89	1.19 ± 0.74^b^	1.35 ± 0.89	1.14 ± 0.79^b^	1.13 ± 0.84^b^
HDL-C (mmol/L)	1.59 ± 0.42	1.56 ± 0.38	1.53 ± 0.39^a^	1.55 ± 0.38	1.54 ± 0.38
LDL-C (mmol/L)	2.89 ± 0.88	2.81 ± 0.84	2.77 ± 0.93	2.32 ± 0.78^b^	2.32 ± 0.82^bd^
Dyslipidemia [n (%)]	528 (35.99)	173 (29.52)^b^	158 (35.59)	130 (19.76)^b^	141 (26.16)^b^

BLF, Bama long-lived families; BNLF, Bama non-long-lived families; BNZLF, Bama non-Zhuang long-lived families; PLF, Pingguo long-lived families; PNLF, Pingguo non-long-lived families; BMI, body mass index; SBP, systolic blood pressure; DBP, diastolic blood pressure; TC, total cholesterol; TG, triglyceride; HDL-C, high-density lipoprotein cholesterol; LDL-C, low-density lipoprotein cholesterol. The values of triglyceride, which were not normally distributed, were log-transformed for analysis but presented as untransformed raw values. The difference between BLF and other group was determined by the Wilcoxon-Mann–Whitney test. ^a^*P* < 0.05 and ^b^*P* < 0.01 in comparison with BLF; ^c^*P* < 0.05 and ^d^*P* < 0.01 in comparison between PNLF and BNLF.

**Table 2:** Comparison of clinical characteristics in the oldest olds from long-lived families

**Parameter**	**BLF (**≥ **90 years)**	**BNZLF (**≥ **90 years)**	**PLF (**≥ **90 years)**
Number (% female)	435 (77.90)	126 (79.40)	144 (61.10) ^b^
Age (years)	93.27 ± 3.05	93.40 ± 2.84	93.68 ± 3.03
Chest (cm)	79.47 ± 7.55	82.12 ± 7.28^b^	80.63 ± 5.86
Waist (cm)	77.69 ± 9.96	78.59 ± 9.66	75.49 ± 7.43^a^
Hip (cm)	84.82 ± 7.00	86.34 ± 8.29^a^	85.35 ± 5.89
BMI (kg/m^2^)	20.52 ± 3.56	21.18 ± 3.88	20.59 ± 3.18
SBP (mmHg)	166.15 ± 27.51	162.35 ± 31.67	166.26 ± 28.57
DBP (mmHg)	90.28 ± 13.35	86.70 ± 14.70^b^	84.40 ± 13.60^b^
TC (mmol/L)	5.19 ± 1.00	5.23 ± 1.17	4.55 ± 0.84^b^
TG (mmol/L)	1.12 ± 0.47	1.25 ± 0.74^a^	0.95 ± 0.38^b^
HDL-C (mmol/L)	1.59 ± 0.38	1.50 ± 0.37^a^	1.57 ± 0.40
LDL-C (mmol/L)	3.09 ± 0.84	3.17 ± 0.97	2.53 ± 0.71^b^
Dyslipidemia [n (%)]	144 (33.10)	52 (41.27)	18 (12.50) ^b^

^a^*P* < 0.05 and ^b^*P* < 0.01 in comparison with BLF.

### Genotypic and allelic frequencies

The genotype and allele frequencies of the polymorphism at −493 MTP locus are shown in Table 3, both of which behaved within the limits of the Hardy-Weinberg law. Overall, the dominant allele and genotype were G and GG, with a frequency of 0.88 and 0.78; respectively. Accordingly, the frequencies of the minor allele, T (0.12) and its homozygotic genotype, TT (0.015) were strikingly lower as compared to G allele and GT/GG genotypes and to most of the reported populations [13-16], demonstrating an evolutionary conservation of the polymorphism in the current tested cohorts. No significant difference in allele and genotype distribution was found across cohorts. However, TT homozygotic genotype seemed to enrich in long-lived families (BLF, BNZLF, and PLF) in comparison with their relevant geographic-matched controls (not significant, *P* > 0.05 for all, Table 3).

**Table 3:** Allelic and genotypic frequency of the MPT −493 polymorphism in 5 families [n (%)]

**Group**	**n**	**Genotype**			**Allele**	
		**GG**	**GT**	**TT**	**G**	**T**
BLF	1467	1126 (76.8)	316 (21.5)	25 (1.7)	2568 (87.5)	366 (12.5)
BNLF	586	480 (81.9)	100 (17.1)	6 (1.0)	1060 (90.4)	112 (9.6)
BNZLF	444	344 (77.5)	90 (20.6)	10 (2.3)	778 (87.6)	110 (12.4)
PLF	658	516 (78.4)	132 (20.1)	10 (1.5)	1164 (88.4)	152 (11.6)
PNLF	539	405 (75.0)	129 (23.9)	5 (0.9)	939 (87.0)	139 (13.0)
Total	3694	2871 (77.7)	767 (20.8)	56 (1.5)	6509 (88.1)	879 (11.9)
*x*^*2*^		13.505			8.577	
*P*		0.096			0.073	

### Genotypes and serum lipid levels

To avoid potential biased results deriving from the extremely small number of MTP TT carriers in each group (Table 3), we analyzed the influence of MTP genotypes on serum lipid levels in two different combined models. First, family combination, i.e. families were combined according to region. As shown in Table 4, the LDL-C levels of the MTP TT carriers were significantly lower than those of GT/GG carriers in all three subgroups (*P* < 0.01 for all); the TC levels of MTP TT carriers were significantly lower than those of GT/GG carriers in the total population and in Bama (*P* < 0.05 for each) but not Pingguo subgroup. When further stratified by gender, the influence of TT genotype on LDL-C but not on TC levels across subgroups remained significant in men (*P* < 0.01), while in females, this influence persisted only in Bama area (*P* < 0.05; Table 4). Second, genotype combination, i.e. individuals with genotype TT and GT were combined on family basis. As shown in Table 5, intra-families comparison revealed no differences in serum concentrations of any lipid parameter within any families between combined group of TT and GT genotypes and GG genotype (*P* > 0.05 for all) except for that the HDL-C levels in the combined group of TT and GT genotypes were significantly lower than in those with the GG genotype in BNZLF (*P* < 0.05). This pattern barely changed at all after sex stratification (data not shown). Inter-families comparison between BLF and the two local controls revealed a few irregular differences which allowed no definite conclusion. However, when it came to the non-local controls, TC, TG and LDL-C levels by (combined) genotypes in BLF were significantly higher than that of the relevant genotypes in PLF and PNLF, in line with observations in Table 1.

**Table 4:** Impact of MTP-493G/T genotype on lipid levels in different area

**Group/genotype**	**n**	**TC**	**TG**	**HDL-C**	**LDL-C**
**Bama**	2497	5.00 ± 1.03^d^	1.28 ± 0.86^d^	1.57 ± 0.41	2.85 ± 0.88^d^
TT	41	4.70 ± 0.89	1.12 ± 0.45	1.44 ± 0.37	2.29 ± 0.77
GT	488	5.07 ± 1.19^a^	1.30 ± 0.92	1.58 ± 0.41^a^	2.89 ± 1.00^b^
GG	1968	4.90 ± 1.00^a^	1.28 ± 0.84	1.58 ± 0.41^a^	2.85 ± 0.85^b^
**Pingguo**	1197	4.38 ± 0.99	1.14 ± 0.81	1.54 ± 0.38	2.32 ± 0.80
TT	15	4.29 ± 1.04	1.04 ± 0.62	1.71 ± 0.49	1.81 ± 0.65
GT	261	4.42 ± 0.94	1.14 ± 0.79	1.55 ± 0.41	2.35 ± 0.77^b^
GG	921	4.37 ± 1.00	1.14 ± 0.82	1.54 ± 0.37	2.32 ± 0.81^b^
**Total**					
TT	56	4.59 ± 0.95	1.10 ± 0.50	1.51 ± 0.42	2.16 ± 0.77
GT	749	4.84 ± 1.15^a^	1.25 ± 0.88	1.57 ± 0.41	2.70 ± 0.95^b^
GG	2889	4.80 ± 1.03^a^	1.23 ± 0.84	1.56 ± 0.40	2.68 ± 0.87^b^
**Bama**/**male**	1413	4.96 ± 1.04^d^	1.37 ± 1.00^c^	1.57 ± 0.44	2.77 ± 0.90^d^
TT	24	4.68 ± 0.95	1.11 ± 0.48	1.43 ± 0.38	2.11 ± 0.78
GT	261	5.01 ± 1.19^a^	1.39 ± 1.02	1.57 ± 0.45	2.80 ± 0.99^b^
GG	1128	4.95 ± 1.00	1.37 ± 0.99	1.56 ± 0.44	2.77 ± 0.87^b^
**Pingguo**/**male**	725	4.46 ± 1.00	1.27 ± 0.93	1.53 ± 0.37	2.35 ± 0.83
TT	11	4.10 ± 0.85	0.91 ± 0.62	1.81 ± 0.53	1.66 ± 0.70
GT	142	4.56 ± 0.95	1.31 ± 0.95	1.57 ± 0.41	2.40 ± 0.79^b^
GG	572	4.44 ± 1.01	1.27 ± 0.93	1.51 ± 0.36^a^	2.35 ± 0.84^b^
**Total/male**					
TT	35	4.49 ± 0.95	1.05 ± 0.53	1.55 ± 0.46	1.97 ± 0.77
GT	403	4.85 ± 1.13^a^	1.36 ± 0.99	1.57 ± 0.44	2.66 ± 0.94^b^
GG	1700	4.78 ± 1.03^a^	1.33 ± 0.97	1.55 ± 0.41	2.63 ± 0.88^b^
**Bama/female**	1084	5.06 ± 1.02	1.16 ± 0.63^d^	1.59 ± 0.36	2.96 ± 0.85
TT	17	4.72 ± 0.84	1.12 ± 0.43	1.46 ± 0.36	2.55 ± 0.71
GT	227	5.13 ± 1.18	1.21 ± 0.80	1.58 ± 0.35	3.00 ± 0.98^a^
GG	840	5.05 ± 0.97	1.15 ± 0.58	1.59 ± 0.37	2.95 ± 0.81^a^
**Pingguo/female**	427	4.25 ± 0.96	0.94 ± 0.52	1.56 ± 0.38	2.26 ± 0.75
TT	4	4.81 ± 1.47	1.39 ± 0.49	1.44 ± 0.18	2.23 ± 0.16
GT	119	4.24 ± 0.90	0.94 ± 0.48	1.53 ± 0.41	2.28 ± 0.74
GG	349	4.25 ± 0.97	0.93 ± 0.53	1.57 ± 0.37	2.26 ± 0.76
**Total/female**					
TT	21	4.74 ± 0.95	1.17 ± 0.45	1.45 ± 0.33	2.49 ± 0.65
GT	346	4.82 ± 1.17	1.12 ± 0.72	1.57 ± 0.37	2.75 ± 0.96
GG	1189	4.82 ± 1.04	1.09 ± 0.58	1.58 ± 0.37	2.75 ± 0.85^a^

^a^*P* < 0.05 and ^b^*P* < 0.01 in comparison with TT genotype of the same group; ^c^*P* < 0.05 and ^d^*P* < 0.01 in comparison between Bama and Pingguo.

**Table 5:** Impact of MTP-493G/T genotype on lipid levels in different families

**Group/genotype**	**n**	**TC**	**TG**	**HDL-C**	**LDL-C**
**BLF**					
GG	1126	5.06 ± 0.99	1.30 ± 0.88	1.59 ± 0.42	2.90 ± 0.85
TT + GT	341	5.08 ± 1.15	1.28 ± 0.91	1.60 ± 0.44	2.85 ± 0.97
**BNLF**					
GG	498	4.89 ± 1.00	1.17 ± 0.70^d^	1.56 ± 0.39	2.78 ± 0.87
TT + GT	88	5.09 ± 0.86	1.25 ± 0.95	1.55 ± 0.34	2.96 ± 0.63^c^
**BNZLF**					
GG	344	4.92 ± 0.97	1.35 ± 0.91	1.55 ± 0.41	2.78 ± 0.81
TT + GT	100	4.83 ± 1.48	1.38 ± 0.86	1.46 ± 0.33^ad^	2.72 ± 1.26
**PLF**					
GG	516	4.39 ± 0.97^d^	1.17 ± 0.82^d^	1.54 ± 0.36	2.32 ± 0.80^d^
TT + GT	142	4.41 ± 0.94^d^	1.04 ± 0.65^d^	1.57 ± 0.43	2.31 ± 0.74^d^
**PNLF**					
GG	405	4.35 ± 1.04^d^	1.10 ± 0.82^d^	1.53 ± 0.37	2.32 ± 0.82^d^
TT + GT	134	4.41 ± 0.94^d^	1.23 ± 0.89	1.55 ± 0.40	2.33 ± 0.80^d^

^a^*P* < 0.05 in comparison with GG genotype of the same group; ^c^*P* < 0.05 and ^d^*P* < 0.01 in comparison with the same genotype between BLF and other groups.

### Correlation between serum lipid parameters and genotypes

Multiple linear regression analysis for the correlation between MTP −493 G/T genotypes and serum lipid parameters is displayed in Table 6. Serum LDL-C level was correlated with genotypes in Bama combined population, BNLF, and the total population (*P* < 0.05 for each) but not in Pingguo populations; serum TC and HDL-C were correlated with genotypes in Bama combined population and BLF; respectively (*P* < 0.05 for each). Serum lipid parameters were also correlated with several environment factors such as age, gender, blood pressure, BMI, and waist circumference in different groups (Table 7).

**Table 6:** Correlation between serum lipid parameters and genotypes in different families

**Lipid**	**Relative factor**	**Unstandardized coefficient**	**Standard error**	**Standardized coefficient**	***t***	***P***
**Bama, all**						
TC	Genotype	0.101	0.048	0.042	2.115	0.035
LDL-C	Genotype	0.098	0.042	0.047	2.343	0.019
**BLF**						
HDL-C	Genotype	0.055	0.026	0.057	2.088	0.037
**BNLF**						
LDL-C	Genotype	0.246	0.100	0.113	2.475	0.014
**BNZLF**						
	Genotype	-	-	-	-	-
**Pingguo**, **all**						
	Genotype	-	-	-	-	-
**PLF**						
	Genotype	-	-	-	-	-
**PNLF**						
	Genotype	-	-	-	-	-
**Total population**						
LDL-C	Genotype	0.075	0.034	0.036	2.186	0.029

BLF, Bama long-lived families; BNLF, Bama non-long-lived families; BNZLF, Bama non-Zhuang long-lived families; PLF, Pingguo long-lived families; PNLF, Pingguo non-long-lived families; TC, total cholesterol; HDL-C, high-density lipoprotein cholesterol; LDL-C, low-density lipoprotein cholesterol.

**Table 7:** Correlation between serum lipid parameters and environmental risk factors in different families

**Lipid**	**Relative factor**	**Unstandardized coefficient**	**Standard error**	**Standardized coefficient**	***t***	***P***
**Bama, all**						
TC	Waist	0.011	0.003	0.115	3.645	0.000
	DBP	0.009	0.002	0.113	5.198	0.000
	Age	0.006	0.001	0.157	6.772	0.000
	Hip	0.019	0.004	0.140	4.811	0.000
	Genotype	0.101	0.048	0.042	2.115	0.035
TG	Waist	0.020	0.003	0.243	7.540	0.000
	Age	−0.005	0.001	−0.161	−6.425	0.000
	DBP	0.005	0.002	0.075	3.336	0.001
	Sex	−0.100	0.037	−0.058	−2.741	0.006
	BMI	0.018	0.007	0.074	2.478	0.013
HDL-C	BMI	−0.015	0.003	−0.129	−5.944	0.000
	Age	0.001	0.000	0.084	3.767	0.000
	DBP	0.002	0.001	0.051	2.208	0.027
LDL-C	Age	0.007	0.001	0.213	9.573	0.000
	Hip	0.017	0.003	0.141	4.977	0.000
	BMI	0.021	0.007	0.081	2.827	0.005
	Sex	0.126	0.037	0.071	3.389	0.001
	DBP	0.005	0.002	0.063	2.842	0.005
	Genotype	0.098	0.042	0.047	2.343	0.019
BLF						
TC	Age	0.006	0.001	0.149	4.812	0.000
	Hip	0.021	0.005	0.150	4.122	0.000
	DBP	0.008	0.002	0.096	3.364	0.001
TG	Sex	−0.117	0.050	−0.066	−2.324	0.020
	Waist	0.017	0.004	0.200	4.907	0.000
	Age	−0.005	0.001	−0.143	−4.243	0.000
	DBP	0.005	0.002	0.074	2.515	0.012
HDL-C	BMI	−0.014	0.003	−0.111	−4.035	0.000
	SBP	0.001	0.000	0.082	2.992	0.003
	Genotype	0.055	0.026	0.057	2.088	0.037
LDL-C	Age	0.008	0.001	0.235	8.931	0.000
	Hip	0.016	0.004	0.137	3.966	0.000
	BMI	0.018	0.009	0.069	1.993	0.046
BNLF						
TC	Waist	0.031	0.004	0.310	7.071	0.000
	Age	0.005	0.002	0.121	2.708	0.007
	Sex	0.354	0.082	0.175	4.330	0.000
	DBP	0.011	0.004	0.135	2.942	0.003
TG	Waist	0.033	0.003	0.430	9.966	0.000
	Age	−0.006	0.001	−0.211	−4.888	0.000
HDL-C	Sex	0.162	0.035	0.207	4.588	0.000
	Age	0.003	0.001	0.224	4.865	0.000
	Waist	−0.011	0.003	−0.277	−3.342	0.001
	Chest	0.013	0.004	0.295	3.646	0.000
	BMI	−0.021	0.009	−0.178	−2.425	0.016
LDL-C	Waist	0.027	0.004	0.318	7.420	0.000
	Age	0.006	0.001	0.177	4.155	0.000
	Sex	0.253	0.069	0.146	3.694	0.000
**BNZLF**						
TC	DBP	0.018	0.004	0.199	4.045	0.000
	Age	0.008	0.002	0.214	4.553	0.000
	Hip	0.022	0.011	0.157	2.081	0.038
	Chest	0.020	0.010	0.152	1.969	0.050
TG	DBP	0.011	0.004	0.158	3.034	0.003
	Age	−0.006	0.002	−0.205	−3.988	0.000
	Waist	0.026	0.005	0.292	5.473	0.000
HDL-C	none					
LDL-C	Age	0.009	0.002	0.281	5.944	0.000
	DBP	0.011	0.004	0.147	2.975	0.003
	Hip	0.028	0.006	0.233	4.877	0.000
	Genotype	0.246	0.100	0.113	2.475	0.014
**Pingguo, all**						
TC	BMI	0.060	0.009	0.214	6.947	0.000
	Age	0.007	0.001	0.193	6.206	0.000
	Sex	−0.153	0.061	−0.079	−2.516	0.012
TG	BMI	0.066	0.008	0.259	8.189	0.000
	Sex	−0.259	0.054	−0.146	−4.781	0.000
	DBP	0.008	0.002	0.113	3.590	0.000
	Age	−0.003	0.001	−0.098	−3.183	0.002
	Waist	0.007	0.002	0.089	2.913	0.004
HDL-C	Age	0.002	0.000	0.149	4.768	0.000
	BMI	−0.012	0.004	−0.106	−3.381	0.001
LDL-C	Age	0.006	0.001	0.181	5.870	0.000
	BMI	0.037	0.007	0.159	5.147	0.000
PLF						
TC	BMI	0.053	0.015	0.182	3.542	0.000
	Age	0.006	0.001	0.168	4.211	0.000
	Chest	0.016	0.006	0.133	2.593	0.010
TG	Waist	0.034	0.004	0.361	8.969	0.000
	Age	−0.005	0.001	−0.146	−3.647	0.000
	Sex	−0.225	0.069	−0.132	−3.279	0.001
HDL-C	SBP	0.002	0.001	0.161	3.858	0000
	Waist	−0.005	0.002	−0.117	−2.811	0.005
LDL-C	BMI	0.053	0.010	0.218	5.396	0.000
	Age	0.005	0.001	0.183	4.534	0.000
**PNLF**						
TC	Age	0.010	0.002	0.199	4.215	0.000
	BMI	0.038	0.013	0.140	2.847	0.005
	DBP	0.009	0.004	0.102	2.059	0.040
TG	BMI	0.070	0.012	0.284	6.005	0.000
	Sex	−0.266	0.085	−0.143	−3.126	0.002
	DBP	0.011	0.004	0.142	2.979	0.003
HDL-C	Age	0.004	0.001	0.206	4.336	0.000
	BMI	−0.013	0.005	−0.120	−2.520	0.012
LDL-C	Age	0.009	0.002	0.232	4.755	0.000
	Sex	−0.162	0.082	−0.096	−1.971	0.049
Total population						
TC	Waist	0.009	0.002	0.090	4.255	0.000
	DBP	0.011	0.002	0.132	7.301	0.000
	Age	0.006	0.001	0.162	8.880	0.000
	BMI	0.040	0.006	0.133	6.453	0.000
TG	BMI	0.043	0.005	0.172	8.309	0.000
	Sex	−0.149	0.030	−0.085	−4.892	0.000
	Waist	0.012	0.002	0.143	6.702	0.000
	Age	−0.004	0.001	−0.120	−6.280	0.000
	DBP	0.007	0.001	0.100	5.447	0.000
HDL-C	Age	0.002	0.000	0.115	5.951	0.000
	BMI	−0.012	0.003	−0.099	−4.576	0.000
	DBP	0.001	0.001	0.042	2.202	0.028
	Waist	−0.002	0.001	−0.045	−2.009	0.045
LDL-C	Age	0.007	0.001	0.021	10.938	0.000
	BMI	0.032	0.005	0.123	6.251	0.000
	DBP	0.006	0.001	0.087	4.698	0.000
	Hip	0.007	0.002	0.069	3.594	0.000
	Sex	0.095	0.031	0.053	3.015	0.003
	Genotype	0.075	0.034	0.036	2.186	0.029

BLF, Bama long-lived families; BNLF, Bama non-long-lived families; BNZLF, Bama non-Zhuang long-lived families; PLF, Pingguo long-lived families; PNLF, Pingguo non-long-lived families; BMI, body mass index; SBP, systolic blood pressure; DBP, diastolic blood pressure; TC, total cholesterol; TG, triglyceride; HDL-C, high-density lipoprotein cholesterol; LDL-C, low-density lipoprotein cholesterol.

## Discussion

In the current study, the frequency of the less-common T variant of MTP gene at −493 locus in the overall population studied is around 0.12, similar to that of other Chinese healthy populations (Shanghai Hans, 0.123 [35]; Hong Konger, 0.126 [11]) and Japanese population (0.17 [36]), but dramatically lower than that of Caucasians (Swedish, 0.25 [13]; Austrian, 0.255 [16]) and African Americans (0.27 [14]), demonstrating a huge frequency gap across races on MTP -493T which might attribute to profoundly different evolutionary pressures at this locus between the East and the West over thousands of years. Intriguingly, however, it is worth noting that this frequency is less than half that of Hei Yi Zhuang (0.26) [15], a Zhuang branch (also termed as Minz) perching on the Sino-Vietnam boundary in Napo County we reported more recently [24]. We have no exact explanations for this great diversity between the two branches basing on the currently available evidence, but factors such as dietary structure and marriage custom might have contributed. On one hand, according to cultural anthropology, the origin of Minzs can at least be dated back to nearly one thousand years ago when Zhuangs immigrated southward to Laos and Thailand [24,37]. Unlike Bama and Pingguo Zhuangs who have more choice on daily staple foods (rice and maize), Minzs have been only growing and living on maize due to adverse mountainous and droughty environments. On the other hand, social isolation, smaller population size (51 800 [15]) and stricter inbreeding of Minzs have resulted in both cultural and genetic homogeneity of this branch relative to other ethnic groups, although signatures of gene mixture with others were also observed [24]. These two crucial selective forces, therefore, may partially account for the higher T allelic frequency of the MTP -493G/T polymorphism in Minz as compared to branches living in Bama and Pingguo area. Alternatively, we could not rule out putative contributions of genetic drift or founder effect on the evolution of this variant in Minz.

Accordingly, owing to relatively lower T allele frequency, MTP TT homogenous individuals were rarely present versus GG and GT in our studying populations. Intriguingly, homozygous TT genotype tended to over-represent in long-lived families both in Bama and Pingguo areas. Furthermore, TT carriers exhibited lower TC and LDL-C concentrations than those with GG genotype, in accordance with data from several previous studies [9-13] but not in others [14-17]. This correlation between genotype and lipoproteins was further supported by subsequent multiple linear regression analysis in some groups (*P* < 0.05) but not in all.

It is clear that the key role of MTP is to transfer lipid to a nascent apoB molecule when it enters the lumen of the ER, mutation in the coding region of the MTP gene leads to undetectable MTP activity and abetalipoproteinemia, a rare autosomal recessive disease with only trace levels of apoB-containing lipoproteins [38]. Moreover, inhibition of MTP by a synthetic MTP-inhibitor lowers atherogenic apoB-containing lipoproteins in patients with homozygous familial hypercholesterolemia [39]. In light of this logic, it is plausible therefore to assume that an increase of MTP gene activity may confer higher levels of apoB-containing lipoproteins. This hypothesis had obtained support in a longitudinal study of young Afro-American men by Juo et al. [14]. However, we observed a reduction rather than an elevation of LDL-C and TC in MTP TT individuals, in agreement with the currently leading results firstly described by Karpe et al. who simultaneously reported that the T allele had an almost 2-fold higher transcriptional activity than did the G allele in vitro [9]. They explained that T allele had fewer but more lipid-rich particles, an increased expression of the MTP gene would lead to a more efficient lipidation of immature VLDL particles. As large VLDLs are not direct precursors of LDL, the input from VLDL to the LDL fraction will decrease and this, in turn, will lower the level of LDL-C in subjects homozygous for the MTP −493 T allele [7,9]. It is also likely that the availability of apoB polypeptides in the ER could restrict the production of apoB-containing lipoprotein, although the -493T up-regulated MTP expression by 2-fold in transfected cells, hence the increased levels of plasma apoB-containing lipoprotein could be disproportional to the increased levels of MTP [14]. More work will be required to discern which of the above explanations is more reasonable. Alternatively, some potentially important factors such as sample sizes, race, age, BMI, and diet should be taken into account, because the interaction between the apoE gene, diet, genetic background, and other covariates has been well documented [2,3,14,40]. Indeed, although our studying cohorts were at large healthy, family-based, well-organized, large sample sizes, and genetically homogeneous, we could not exclude the putative effects of diet on lipid variables, which was an unexpected finding in the current study.

As can be seen in Tables 1, 2, and 4, serum TC, TG and LDL-C levels and the prevalence of dyslipidemia were higher in Bama populations including nonagenarian subgroup as compared to non-local controls, Pingguo populations, albeit a few heterogeneities were also observed among Bama subgroups. This is somewhat beyond our theoretical predictions because almost all previous studies by other investigators had addressed that Bama long-lived individuals exhibited superior lipid profiles, lower morbility and better survivorship relative to general populations inside or outside Bama area [41,42]. Our subsequent nutrition survey indicated that inhabitants in both Bama and Pingguo areas have experienced radical changes in diet and lifestyle in the past decade due to rapidly socioeconomic improvement (data not shown). We noted, in particular, that individuals from Bama area tend to intake more fats, more proteins, more carbohydrates, and even more alcoholic beverages, which generate more calories than did before (data not shown). Furthermore, Bama families usually consume cheap fat pork and cook foods using animal oil (lard oil), while Pingguo families, with better socioeconomic status, usually consume expensive lean pork and vegetable oil (mainly peanut and camellia oil). It is widely recognized that there are great heterogeneities in composition, especially fatty acid between vegetable oil and lard oil and the former contains more unsaturated fatty acids which are favorable for health. Alternatively, a diet rich in saturated fat and cholesterol was found to promote a high-absorption status in GTP TT French women recently [2]. Therefore, these radical dietary changes and potential gene-diet interaction may play critical role in shaping the current lipoprotein profiles in Bama cohorts. Further studies are necessary to clarify this point.

Geesaman and colleagues were the first to link MTP gene with exceptional human longevity. They found that a two-marker haplotype composed of the rs2866164-G/MTP-Q95 (rs61733139) was significantly underrepresented in a sample of 190 long-lived Americans of European extraction, and suggested that this risk haplotype confers a higher mortality earlier in life and consequently an extended life expectancy [3]. However, these findings have not since been validated in other long-lived cohorts from French [3], German [19], Danish [5], Dutch [20], British [43], and Italian populations [21], leading several to conclude that the findings of Geesaman and colleagues were confounded by population stratification in their controls [19,22]. Conversely, Huffman et al. [22] uncovered most recently that MTP rs2866164-CC instead of -GT/GG served as a deleterious but not a longevity genotype. It underrepresented with aging before 85 years old but sharply enriched in nonagenarians and centenarians wherein its unfavorable effects might be buffered by other longevity genotypes such as CETP (rs5882), ApoC3 (rs2542052), AdipoQ (rs56354395). MTP -493G/T (rs1800591) had been found to be in perfect linkage disequilibrium with rs2866164 [3,19], and the minor T of -493G/T described in this article corresponds with the minor allele G of rs28166164 [3,20]. Here, the allelic and genotypic frequencies of MTP -493G/T did not differ significantly between BLF and other tested cohorts, suggesting a lack of association between this polymorphism and human longevity in Bama area, lending further support to the leading hypothesis on this point. However, the TT genotype tended to overrepresent in the three long-lived families as compared to their geographic-matched controls. Together with the finding that TT individuals exhibited lower LDL and TC levels than did G carriers, it appears that T variant plays a favorable role in the lipid modulation in our tested cohorts. However, we could not infact rule out the possibility that T is a risk allele as proposed by Geesaman et al. [3]. Given this is true, the enrichment of TT in long-lived families could be buffered by unknown longevity genotypes. To clarify these points, further works, e.g. functional analyses, buffering gene determinations, longitudinal studies, are necessary.

## Conclusion

No direct association between MTP -493G/T and longevity was detected in Bama Zhuang populations. However, the facts that MTP −493 TT genotype tends to enrich in long-lived families and individuals harboring TT genotype display lower LDL and TC levels suggest that MTP -493G/T polymorphism may influence the aging outcome of Bama population by modulating the lipid profiles and the onset of aged-related diseases. Alternatively, the radical dietary changes may be another important determinant for lipid profiles and a big challenge for the longevity in Bama area, thus public health education under modern lifestyles need to be strengthened in Bama populations.

## Competing interests

The authors declare that they have no competing interests.

## Authors’ contributions

SLP participated in the design, undertook genotyping, and drafted the manuscript. XQL, SHL, CWL, MY, LLD, ZS, HYW, JBH and JHP helped with genotyping. ZPL, HL, CYH and GFP took part in the epidemiological survey and collected the samples. RXY and JHP conceived the study, participated in the design, carried out the epidemiological survey, collected the samples, and helped to draft the manuscript. All authors read and approved the final manuscript.
